# Palestinian physicians’ self-reported practice regarding antibiotic use for upper respiratory tract infections in primary healthcare

**DOI:** 10.3389/fmed.2023.1139871

**Published:** 2023-03-30

**Authors:** Bessan Maraqa, Zaher Nazzal, Suha Hamshari, Nardine Matani, Yasmeen Assi, Mousa Aabed, Furqan Alameri

**Affiliations:** ^1^Family and Community Medicine Department, College of Medicine, Hebron University, Hebron, Palestine; ^2^Consultant Community Medicine, Faculty of Medicine and Health Sciences, An-Najah National University, Nablus, Palestine; ^3^Consultant Family Medicine, Department of Family and Community Medicine, Faculty of Medicine and Health Sciences, An-Najah National University, Nablus, Palestine; ^4^Faculty of Medicine and Health Sciences, An-Najah National University, Nablus, Palestine; ^5^Consultant Family Medicine, Ministry of Health, Gaza, Palestine; ^6^Ministry of Health, Alhussein Teaching Hospital, Karbala, Iraq

**Keywords:** primary care, antibiotics, family medicine, West Bank and Gaza, upper respiratory tract infections

## Abstract

**Background:**

The main objective of this study was to evaluate the antimicrobial therapy knowledge, attitudes, and practices of primary care physicians in the West Bank and Gaza.

**Methods:**

Between January and April 2021, this cross-sectional survey was conducted. A link to the online survey was sent *via* confidential email lists to 336 primary care physicians who treated patients in Ministry of Health clinics. The survey questions scoring system was devised in order to evaluate the physicians’ practice, knowledge, and attitudes. Our scoring system identified favorable (good) and unfavorable (average and poor) antibiotic practices. In addition to independent *t*-test, the Chi-square test was used to compare the two groups of physicians’ knowledge, attitudes, and practices with their background characteristics. A multivariate analysis was performed to identify potential confounding variables having significant relationships.

**Results:**

Of the 336 distributed questionnaires, 316 were completed, with a response rate of 94%. More than half (54.7%) were males, half (51.6%) were between 30 and 45 years of age, and most were general practitioners (74.1%). The overall good knowledge and attitude scores were 125 (39.6%) and 194 (61.4%), respectively. More than half (58.2%) had good antibiotic prescription practices. Females reported significantly more favorable practices than males, as did family medicine specialists compared with general practitioners. Finally, knowledge about antibiotic prescriptions had a substantial impact on changing their practices. However, physicians’ attitudes toward antibiotic prescriptions did not have a significant role in shaping their practices.

**Conclusion:**

Overuse of antibiotics is a significant health issue in Palestine and worldwide. Most physicians know that improper antibiotic usage can cause antimicrobial resistance. More than two-thirds avoided needless antimicrobial prescriptions. In practicing antimicrobial stewardship, most prescribed fewer antibiotics and did not prescribe because of patient pressure. Family medicine specialists, female doctors, and those with high knowledge scores regardless of caseload were more likely to have good practices.

## Introduction

Antibiotics are generally considered safe drugs, they are prescribed to relieve symptoms of bacterial infection, particularly in primary care, and they are used in the treatment of various diseases such as acute otitis media, group A beta-hemolytic streptococcal pharyngitis, epiglottitis, and bronchitis (1). Despite having low cost and being readily available, global concerns regarding antibiotic usage exist (2).

Overuse of antibiotics is a significant health issue worldwide; between 20 and 30% of antibiotic use is believed to be ineffective or useless (3). Antibiotic resistance is a globally growing issue (4), and this issue has increased the risk of morbidity and mortality. For example, in Europe, 25,000 deaths have been recorded (5) due to infection with antibiotic-resistant microorganisms.

Upper respiratory tract infections (URTI) which are defined as the nose, pharynx, larynx, and trachea subglottic infections, are known to be the most common cause of inappropriate antibiotic use and are a common reason for primary care consultations (6). Although most URTIs are viral in origin, antibiotic prescriptions for the treatment of URTI have increased in recent years, with World Health Organization (WHO) reporting prescription increases from 43 to 71% between 1982 and 2006 (7). This makes the use of antibiotics inappropriate and ineffective (8). Moreover, the improper use of antibiotics in viral illnesses increases the likelihood of inadequate response to regular therapy in antibiotic-resistant patients, causing contagion for longer periods of time, and placing strain on healthcare systems (9).

Despite global awareness, the prevalence of antibiotic resistance in some countries is still not fully recognized by some physicians (10, 11); furthermore, some physicians continue to prescribe antibiotics even though they know it is not necessary (12). It is believed that doctors’ decision to prescribe antibiotics could be affected by patients perception of good quality care; thus, some physicians tend to respond to patients’ pressure to avoid their dissatisfaction with the care they receive (10). In addition, in many countries, including Palestine, patients can receive antibiotics from pharmacists without a prescription.

Our literature review has shown that there is no official assessment of primary care physicians’ knowledge, attitudes, and practices regarding antimicrobial therapy in the occupied West Bank Territories and Gaza. This study aimed to address this gap across the Occupied West Bank Territories and Gaza.

## Methods

### Study design and population

This cross-sectional study was carried out at the Primary Healthcare centers (PHC) in the Occupied West Bank and the Gaza strip from January to April 2021. Ministry of Health’s Primary Healthcare centers are distributed throughout the country and serve as the patients’ first point of contact with the healthcare system. The study included all PHC physicians who worked at these centers. We excluded physicians who work in administrative positions and do not treat patients. An online survey link was sent through closed email lists to 336 primary care physicians.

The sample size was calculated using the formula: [n = Z^2^*P*(1-P)/d^2^], where *Z* is the confidence level statistic (*Z* = 1.96), P is the expected proportion of subjects with good practice, and d is the precision. A sample size of 245 was calculated to be the minimum required, assuming that 20% of primary care physicians have good practices (13), and a 95% confidence interval (CI), with 0.05 absolute precisions on either side of the predicted proportion. We inflated the number to 336 to account for an expected non-response rate of 40% among participants (14).

All procedures in this study were carried out in accordance with the Helsinki Declaration. The study was approved by the Institutional Review Board of An-Najah National University (Med. Nov. 2020/16).

The Palestinian Ministry of Health’s Primary Healthcare Department granted permission to conduct the study, and participants were approached and invited to participate voluntarily. We accompanied the questionnaire with a cover letter highlighting the objectives of the study and assuring respondents that their responses would be kept strictly confidential and anonymous. An online informed consent to participate was obtained from all participants by asking them to confirm their agreement with the information provided and their willingness to participate online by tapping the button “I agree.”

### Measures

The questionnaire was based on extensive literature review (2, 15) and consisted of 40 items with 4 sections: (1) demographic and practice data (8 questions, with a question asking how many prescriptions for antibiotics were written in the last 7 days); (2) antibiotic prescriptions practices (10 questions with yes/no responses); (3) assessing the physicians’ knowledge (4 questions with yes/no responses and 4 scenarios with appropriate/unsure/not appropriate responses); and (4) attitude assessment (9 questions with 4-point Likert scale responses from strongly agree to strongly disagree).

We pre-tested the questionnaire to ensure its validity and reliability. For content validity, three experts in the field reviewed it, and then we translated it into Arabic. A pilot study on a sample of 19 physicians checked the tool’s simplicity and readability, and the time needed for completion. The necessary adjustments were made to finalize the questionnaire.

A scoring system was created to assess the physician’s practice, knowledge, and attitudes. The correct answers were summed to get the final score for each participant. The practice score ranged between 0 and 10. Physicians with good practice received scores ranging from 8 to 10, average practice ranged from 6 to 8, and poor practice scored less than 6. The total knowledge score ranged from 0 to 12. Greater than 10 was deemed to be good knowledge; 8 to 10 was average knowledge; and less than 8 was poor knowledge. Each of the four clinical case scenarios received a score of 0 for an incorrect response, 2 for correct, and 1 for unsure. The total score for attitudes ranged from 9 to 36. The total attitude score was divided into three levels based on Bloom’s cutoff point (16); a score of 29 or greater was considered good or appropriate attitudes, 22 to 29 average attitudes, and less than 22 poor attitudes. The primary study outcome, practice, was dichotomized as either favorable or unfavorable. The favorable practice group represents Physicians’ with good practice, whereas the unfavorable practice group represents Physicians’ with average or poor practice.

### Data analysis

We used IBM SPSS Statistics for Windows, version 21 (IBM Corp., Armonk, NY, United States) for data analysis. A two-tailed *p* < 0.05 indicated statistical significance. The Kolmogorov–Smirnov test was utilized to ascertain the data’s normality; the results confirmed that the data followed a normal distribution. Frequencies, percentages, and mean ± standard deviation (SD) were used to describe physicians’ characteristics. We used Chi-square and the independent *t*-test to compare the physicians’ knowledge, attitudes, and practice with their between different groups background characteristics and the independent *t*-test, where applicable. Finally, we employed multivariate analysis using binary logistic regression was conducted to track possible confounders of significant associations.

## Results

A total of 336 physicians received the questionnaire and 316 responded with a 94% response rate. Table 1 shows the physicians’ demographic characteristics. Males represented 54.7% of the respondents (173). Half (163, 51.6%) of all respondents were between 30 and 45 years of age. Most respondents were general practitioners (74.1%). The majority of the physicians (69.0%) treated more than 30 patients per day, with half (50.9%) having 5 to 19 years of practice experience (see Table 1).

**Table 1:** Sociodemographic characteristics of the studied sample (*n* = 316).

Variable	Frequency	Percentage
District (West Bank and Gaza)		
North	109	34.5
Middle	57	18.0
South	148	46.8
Age (years)		
Less than 30	76	24.1
30–45	163	51.6
More than 45	77	24.4
Specialty		
General practitioner (GPs)^*^	234	74.1
Family medicine	41	13.0
Others	41	13.0
Gender		
Female	143	45.3
Male	173	54.7
Social status		
Unmarried	52	16.5
Married	263	83.2
Average number of patients\day		
Less than 30	98	31.0
More than 30	218	69.0
Experience		
Less than 5 years	98	31.0
5–19 years	161	50.9
More than 20 years	56	17.7
Prescriptions written in the last (7 days)		
0	34	10.8
1–5	117	37.0
6–10	75	23.7
More than 10	90	28.5

^*^GPs complete one year of internship after medical school.

### Knowledge

Table 2 shows physicians’ knowledge about antibiotic use. Most doctors knew antibiotics are not used for viral infections (287, 90.8%) and that antibiotics do not reduce symptoms (224, 70.9%). Most physicians knew why antibiotics resistance occurs (299, 94.6%), and self-treatment and antibiotic misuse are the most important factors (307, 97.2%). Clinical case scenarios were used to check the knowledge of the physicians. For example: Doctors were asked in which clinical cases they would prescribe antibiotics. The majority responded that it is incorrect to prescribe antibiotics to smokers with yellow sputum who had no fever (264, 83.5%), and only (4.4%) were unsure. The overall knowledge score on the four clinical case scenarios was: 125 (39.6%) good, 101 (32.0%) average, and 90 (28.5%) poor.

**Table 2:** Physician responses to questions about knowledge toward antibiotics.

Statements	Yes	No	
Antibiotics are used for the treatment of viral infections	29 (9.2%)	287 (90.8%)	
Antibiotic treatment reduces the severity of URTIs	92 (29.1%)	224 (70.9%)	
Antibiotic resistance occurs when a bacteria’s sensitivity to antibiotics decreases	299 (94.6%)	17 (5.4%)	
Self-treatment and antibiotic misuse are two of the most important causes of antibiotic resistance	307 (97.2)	9 (2.8%)	
In which of the following situations would an antibiotic prescription be appropriate?	Correct	Incorrect	Unsure
A 40-year-old smoker who coughs up yellow sputum, without fever, normal CXR	38 (12%)	264 (83.5%)	14 (4.4%)
A 25-year-old, complaining of acute sinusitis for a few days without fever, and examination shows tenderness over the anterior sinuses area	192 (60.8%)	103 (32.6%)	21 (6.6%)
A 17-year-old complaining of sore throat, nonproductive cough, Temp: 38, and exam shows tonsillar congestion with no palpable lymph nodes	137 (43.4%)	157 (49.7%)	22 (7%)
A pregnant woman given Levofloxacin for acute pneumonia	42 (13.3%)	244 (77.2%)	30 (9.5%)
Overall knowledge score			
Good	125 (39.6%)		
Average	101 (32.0%)		
Poor	90 (28.5%)		

### Attitudes

Table 3 shows the physicians’ attitudes about antibiotic use. Over half (58.2%) strongly disagreed that a bacteria’s resistance was unaffected when used to treat respiratory infection. Almost half of the physicians did not prescribe one or more antibiotics to cover all pathogens for a non-confirmed diagnosis (145, 45.9%), or if they did not have time for a clinical examination (229, 72.5%), or did not have time to explain the disease’s possible causes (227, 71.8%). Most would not prescribe antibiotics to improve their patients’ trust in their skills (260, 82.3%). Over half agreed or strongly agreed (56.6%) that patients who thought they needed antibiotics would get them from a pharmacy even if a doctor did not prescribe them. The total scores of attitudes toward antibiotics were good (194, 61.4%), average (116, 36.7%), and poor (6, 1.9%).

**Table 3:** Physicians’ attitudes about antibiotic use.

Attitude statements	Strongly agree	Agree	Disagree	Strongly disagree
Antibiotics do not affect a bacteria’s resistance when they are used for respiratory infections.	12 (3.8%)	36 (11.4%)	84 (26.6%)	184 (58.2%)
Antibiotic resistance can be managed by the use of new antibiotics.	25 (7.9%)	80 (25.3%)	94 (29.7%)	117 (37%)
When the diagnosis is not confirmed, it’s better to give one or more antibiotics to cover all possible pathogens.	16 (5.1%)	55 (17.4%)	100 (31.6%)	145 (45.9%)
Antibiotics are prescribed when there is a potential risk that a bacteria pathogen is causing the respiratory infection.	44 (13.9%)	125 (39.6%)	96 (30.4%)	51 (16.1%)
When I do not have time for a clinical evaluation, I prescribe an antibiotic for a respiratory infection.	3 (0.9%)	19 (6%)	65 (20.6%)	229 (72.5%)
I’ll occasionally prescribe an antibiotic to increase a patient’s confidence in my medical skills.	2 (0.6%)	12 (3.8%)	42 (13.3%)	260 (82.3%)
If I do not have time to explain the disease’s possible causes, I’ll prescribe an antibiotic and schedule an appointment for a follow-up.	5 (1.6%)	27 (8.5%)	57 (18%)	227 (71.8%)
If the patient believes he needs an antibiotic for respiratory symptoms, he will get it from the pharmacy on his own even if I do not prescribe it	100 (31.6%)	79 (25%)	47 (14.9%)	90 (28.5%)
Antibiotics for respiratory infections should only be recommended by a doctor	249 (78.8%)	52 (16.5%)	8 (2.5%)	7 (2.2%)
Overall attitude score				
Good	194 (61.4%)			
Average	116 (36.7%)			
Poor	6 (1.9%)			

### Practice

Table 4 shows physicians’ antibiotic prescription practice. Most of the physicians (305, 96.5%), indicated that guidelines influence their selection of prescribed antibiotics, and prescribe no more than one antibiotic at a time (292, 92.4%). The majority indicated that they do not feel pressured by patients or family members to prescribe antibiotics (286, 90.5%), do not prescribe antibiotics to prevent secondary infection (189, 59.8%), or to patients with suspected COVID-19 (179, 56.9%). Practice scores were: 184 (58.2%) good, 119 (37.7%) average, and 13 (4.1%) poor.

**Table 4:** Physicians’ responses to statements about antibiotic prescription practice.

Statements	Yes	No
I usually prescribe antibiotics based on guidelines	305 (96.5%)	11 (3.5%)
To control the disease, I usually prescribe two or three antibiotics.	24 (7.6%)	292 (92.4%)
When a family member or a patient insists on antibiotics, I prescribe them.	30 (9.5%)	286 (90.5%)
I usually prescribe antibiotics to avoid secondary bacterial infection.	127 (40.2%)	189 (59.8%)
Antibiotic prescriptions are influenced by the economic condition of my patients	19 (61.1%)	123 (38.9%)
I usually antibiotics prescribed under their generic name	165 (52.2%)	151 (47.8%)
I prescribe antibiotics dosage based on the patient’s weight and age.	306 (96.8%)	10 (3.2%)
I always describe the duration of treatment when prescribing antibiotics.	302 (95.6%)	14 (4.4%)
Regardless of symptoms, I advise the patient about the necessity of completing the prescribed medication period.	299 (94.6%)	17 (5.4%)
I prescribed antibiotics to suspected COVID-19 patients.	137 (43.4%)	179 (56.6%)
Overall practice score		
Good	184 (58.2%)	
Average	119 (37.7%)	
Poor	13 (4.1%)	

The statistically significant associations identified between favorable antibiotics prescription practices included: specialty, gender, and marital status. Family medicine specialists had a more favorable practice than GP (value of *p* = 0.007). Likewise, female physicians showed favorable practice than males (value of *p* = 0.026). Higher scores on attitudes and knowledge were associated with favorable practice and not related to the number of patients seen in a day or years of experience. See Table 5.

**Table 5:** Determinants of favorable practice.

	Favorable practice (*n* = 184)	Unfavorable practice (*n* = 132)	*p*-value
District			
North	55 (50.5%)	54 (49.5%)	
Middle	38 (66.7%)	19 (33.3%)	0.092
South	90 (60%)	58 (39.2%)	
Age (years)			
<30	43 (56.6%)	33 (43.4%)	
30–45	98 (60.1%)	65 (39.9%)	0.777
>45	43 (55.8%)	34 (44.2%)	
Specialty			
General practitioner (GPs)	127 (54.3%)	107 (45.7%)	
Family medicine	33 (80.5%)	8 (19.5%)	0.007
Others^†^	24 (58.5%)	17 (41.5%)	
Gender			
Female	93 (65.0%)	50 (35.0%)	
Male	91 (52.6%)	82 (47.4%)	0.026
Marital status			
Unmarried	32 (61.5%)	20 (38.5%)	0.001
Married	152 (57.8%)	111 (4.2%)	
Average patients treated per day			
Less than 30	59 (60.2%)	39 (39.8%)	0.633
More than 30	125 (57.3%)	93 (42.7%)	
Experience			
<5 years	58 (59.2%)	40 (40.8%)	
5–19 years	89 (55.3%)	72 (44.7%)	0.483
≥20 years	36 (64.3%)	20 (35.7%)	
How many prescriptions for antibiotics have you written in the last 7 days?			
0	22 (64.7%)	12 (35.2%)	
1–5	77 (65.8%)	40 (34.2%)	0.073
6–10	41 (54.7%)	34 (45.3%)	
>10	44 (48.9%)	46 (51.1)	
Overall knowledge			
Good	92 (73.6%)	33 (26.4%)	
Average	52 (51.5%)	49 (48.5%)	<0.001
Poor	40 (44.4%)	50 (55.6%)	
Overall attitude			
Good	129 (66.5%)	65 (33.5%)	
Average	53 (45.7%)	63 (54.3%)	0.001
Poor	2 (33.3%)	4 (66.7%)	

Multivariate logistic regression confirmed the earlier findings that Family Medicine specialists reported favorable practice than GPs (*p* value = 0.038), and females reported significantly favorable practice than males (value of *p* = 0.037). Finally, knowledge about antibiotic prescriptions showed a significant role in shaping favorable practice (value of *p* = 0.002) (Table 6). On the other hand, physicians’ attitudes about antibiotics prescriptions did not have a significant role in shaping their practice (value of *p* = 0.079).

**Table 6:** Factors significantly associated with Physicians’ favorable practice.

Domain	Β	SE	Adjusted OR	*p*-value	95%CI of adjusted OR
Specialty					
GP	0.929	0.448	2.5	0.038	1.1–6.1
FM Specialist	0.202	0.364	1.2	0.579	0.60–2.5
Others					
Sex					
Female	0.537	0.258	1.7	0.037	1.1–2.8
Male^1^					
Attitude					
Average	0.878	0.912	2.4	0.335	0.40–14.4
Good	1.586	0.903	4.9	0.079	0.83–28.7
Poor					
Knowledge					
Average	0.029	0.308	1.0	0.925	0.56–1.9
Good	0.967	0.312	2.6	0.002	1.4–4.800
Poor^1^					

## Discussion

This is the first study assessing the knowledge, attitudes and practices regarding the use of antibiotics by primary care physicians in the Ministry of Health clinics in the West Bank and Gaza. This is an important because physicians in these areas practice without clear antibiotic prescribing guidelines or specific restrictions.

Almost 40% demonstrated good knowledge about antibiotics usage. It is worth mentioning that 60.8% agreed incorrectly with prescribing antibiotics for acute sinusitis after a few days without fever. Our findings suggest that Palestinian primary care physicians need educational initiatives to improve their knowledge about the appropriate use of antibiotics. Studies have shown that educational interventions improve practice, specifically 62% of studies reviewed in a systematic review showed improved guideline adherence, decreased total number of antibiotic prescriptions, improved physicians’ attitudes and behaviors, and improved pharmacy practice related to antibiotic prescriptions (17).

Attitudes and reported practice by Palestinian primary care physicians were only slightly better, 61.4 and 58.2%, respectively. Appropriate attitudes demonstrated no significant association favorable practice.

Public advocacy and education about over prescribing antibiotics and the detrimental effects will play a crucial role in increasing physicians’, all health professionals’, and patients’ knowledge of antibiotics usage (18). In order to address the educational needs of physicians, the creation of evidence-based antibiotic prescribing recommendations endorsed by relevant national/regional medical organizations should be a priority. These guidelines should be extensively publicized to physicians and pharmaceutical firms, and will promote prudent prescribing of antimicrobials and reduce antibiotic misuse (19).

However, in the Palestinian community, pharmacists play a role in influencing patients’ decisions about antibiotics. It is concerning that most physicians agreed that patients will get antibiotics for viral infections from a pharmacy on their own even if not prescribed. This is not only a Palestinian problem as other countries such as Saudi Arabia have similar findings (2).

Our finding that the majority of physicians refused to prescribe antibiotics to increase patients’ confidence in their medical practice is positive as another study in the governmental hospitals outpatient clinics in the northern occupied Palestinian territories (20) found that patient demand for antibiotics contributed to antibiotic misuse in order to assure patient satisfaction with their care.

Favorable antibiotic prescription practice was significantly different between family medicine specialists and GPs or practitioners from other specialties. This suggests the value of the family medicine specialty, particularly in middle-income countries such as Palestine, where this specialty is relatively new. More family medicine trained physicians can improve the quality of care and it is important to support and empower them in sharing their knowledge with GPs and other colleagues (18).

Furthermore, it is notable that there was a statistically significant difference in favorable practice between females and males. This could be attributable to the fact that females make up the majority of family medicine physicians in Occupied Palestine and Gaza. Other factors such as age, district, and years of practice did not have an impact on a physician’s practice in our study. This is consistent with the findings in studies done in Saudi Arabia, Scotland, and France (2, 15). In contrast, a study conducted in Indonesia showed that physicians with less than 7 years of practice experience were less likely to prescribe antibiotics (21). Hence, younger physicians had more contemporary training. Since physicians in Occupied Palestine and Gaza are not required to do continuing medical education, they are more likely to practice medicine that is not up to date (22–24). This underlines the importance of implementing guidelines that are continually improved and to require some kind of practice improvement activities.

Finally, previous researchers have linked a high number of patients treated daily to high antibiotic prescription rates. This has been linked to time pressures or decision fatigue (22). Surprisingly, our research found no link between these two variables. It may be that participants attempted to respond in a more socially acceptable manner, or because the small sample size did not reflect the correct rate (25).

The study limitations include: The self-report nature of the questionnaire allowed more socially acceptable responses instead of reporting actual practice. While we ensured confidentiality in an attempt to reduce the self-report bias, the option of completing the questionnaire online or on paper may have affected the degree of honesty, but was done to improve completion rate. The United Nations Relief and Works Agency (UNRWA) clinics were not included in our sample so our findings cannot be generalized to the GPs practicing in that setting. We chose Ministry of health (MOH) clinics on the West Bank and the Gaza Strip to understand what government sector sponsored activities are needed to improve antibiotic use.

## Conclusion

Most of the physicians were knowledgeable about what constitutes improper use of antibiotics and how it can, among other factors, contribute to antimicrobial resistance. More promising figures were seen in physicians’ attitudes to prescribing with more than two-thirds avoiding unnecessary antimicrobial prescriptions and when antibiotics are not indicated. A similar pattern was also seen in practicing antimicrobial stewardship where the majority used a conservative approach to the number of antibiotics prescribed or did not prescribe because of patient pressure. Of note, good practice was more likely seen in family medicine specialists, female doctors, and those who scored high in knowledge questions regardless of caseload.

## Data availability statement

The original contributions presented in the study are included in the article/Supplementary material, further inquiries can be directed to the corresponding author.

## Ethics statement

All methods involving human participants in this study were conducted per ethical research standards. The study was conducted in conformity with the ethical norms of An-Najah National University (ANNU). The Ministry of Health approved authorization for the study to be conducted in PHC settings, and participants were approached and invited voluntarily to participate. Participants were assured of their confidentiality and anonymity. This study was performed in accordance with the ethical standards of the institutional research committee and with the 1964 Helsinki Declaration and its later amendments or comparable ethical standards. It was approved by the Institutional Review Board (IRB) of An-Najah National University.

## Author contributions

BM: designing the work, conducting data analyses and interpretations, drafting the work, and final approval of the published version. ZN: analysis and interpretation of data, drafting of the work, and final approval of the published version. SH: designing the work, drafting the work, final approval of the published version, and agreement to be accountable for all aspects of the work, including ensuring that any questions about the work’s accuracy or integrity are appropriately investigated and resolved. YA: acquisition of data, drafting of the work, and final approval of the published version. NM: acquisition of data, drafting of the work, and final approval of the published version. MA: data collection and drafting of the work. FA: drafting of the work and final approval of the published version. All authors contributed to the final manuscript’s development and approval.

## Conflict of interest

The authors declare that the research was conducted in the absence of any commercial or financial relationships that could be construed as a potential conflict of interest.

## Publisher’s note

All claims expressed in this article are solely those of the authors and do not necessarily represent those of their affiliated organizations, or those of the publisher, the editors and the reviewers. Any product that may be evaluated in this article, or claim that may be made by its manufacturer, is not guaranteed or endorsed by the publisher.
