# Supplementary material for: Palestinian physicians’ self-reported practice regarding antibiotic use for upper respiratory tract infections in primary healthcare
Source: Front Med (Lausanne). 2023 Mar 30;10:1139871. doi: 10.3389/fmed.2023.1139871 (PMC10098349; doi:10.3389/fmed.2023.1139871)
Supplement: Supplementary file 1 [file Table_1.DOCX]

Appendix

# Would you like to participate in this study?

- Yes
- No

# Sociodemographic characteristics: -

| District (West Bank and Gaza)  - North - Middle - South | Social status  - Unmarried - Married |
| --- | --- |
| Age (years): ------- | Number of patients\day: ------- |
| Specialty  - General practitioner (GPs) - Family medicine - Others | Experience (years): -------- |
| Gender  - Female - Male | Prescriptions written in the last (7 days)  - 0 - 1-5 - 6-10 - More than 10 |

# Medical Knowledge: -

## Please indicate your agreement or disagreement with each statement below: -

| Statement | Strongly agree | Agree | Disagree | Strongly disagree |
| --- | --- | --- | --- | --- |
| Antibiotics are used for the treatment of viral infections |  |  |  |  |
| Antibiotic treatment reduces the severity of URTIs |  |  |  |  |
| Antibiotic resistance occurs when a bacteria's sensitivity to antibiotics decreases |  |  |  |  |
| Self-treatment and antibiotic misuse are two of the most important causes of antibiotic resistance |  |  |  |  |

## In which of the following situations would an antibiotic prescription be appropriate?

| Situation | Correct behavior | Incorrect behavior | Unsure |
| --- | --- | --- | --- |
| A 40-year-old smoker who coughs up yellow sputum, without fever, normal CXR |  |  |  |
| A 25-year-old, complaining of acute sinusitis for a few days without fever, and examination shows tenderness over the anterior sinuses area |  |  |  |
| A 17-year-old complaining of sore throat, nonproductive cough, Temp: 38, and exam shows tonsillar congestion with no palpable lymph nodes |  |  |  |
| A pregnant woman given Levofloxacin for acute pneumonia |  |  |  |

## Please indicate your agreement or disagreement to the following statements about your attitude to the antibiotic usage:

| Statement | Strongly agree | Agree | Disagree | Strongly disagree |
| --- | --- | --- | --- | --- |
| Antibiotics do not affect a bacteria's resistance when they are used for respiratory infections. |  |  |  |  |
| Antibiotic resistance can be managed by the use of new antibiotics. |  |  |  |  |
| When the diagnosis isn't confirmed, it's better to give one or more antibiotics to cover all possible pathogens. |  |  |  |  |
| Antibiotics are prescribed when there is a potential risk that a bacteria pathogen is causing the respiratory infection. |  |  |  |  |
| When I don't have time for a clinical evaluation, I prescribe an antibiotic for a respiratory infection. |  |  |  |  |
| I'll occasionally prescribe an antibiotic to increase a patient's confidence in my medical skills. |  |  |  |  |
| If I don't have time to explain the disease's possible causes, I'll prescribe an antibiotic and schedule an appointment for a follow-up. |  |  |  |  |
| If the patient believes he needs an antibiotic for respiratory symptoms, he will get it from the pharmacy on his own even if I do not prescribe it |  |  |  |  |
| Antibiotics for respiratory infections should only be recommended by a doctor |  |  |  |  |

# Clinical Practice:

## Please indicate your agreement or disagreement to the following statements about antibiotic prescription practice:

| Statement | Strongly agree | Agree | Disagree | Strongly disagree |
| --- | --- | --- | --- | --- |
| I usually prescribe antibiotics based on guidelines |  |  |  |  |
| To control the disease, I usually prescribe two or three antibiotics. |  |  |  |  |
| When a family member or a patient insists on antibiotics, I prescribe them. |  |  |  |  |
| I usually prescribe antibiotics to avoid secondary bacterial infection. |  |  |  |  |
| Antibiotic prescriptions are influenced by the economic condition of my patients |  |  |  |  |
| I usually prescribed antibiotics under their generic name |  |  |  |  |
| I prescribe antibiotics dosage based on the patient's  weight and age. |  |  |  |  |
| I always describe the duration of treatment when prescribing antibiotics. |  |  |  |  |
| Regardless of symptoms, I advise the patient about the necessity of completing the prescribed medication period. |  |  |  |  |
| I prescribed antibiotics to suspected COVID-19 patients |  |  |  |  |
